# *Limnotrachelobdella hypophthalmichthysa* n. sp. (Hirudinida: Piscicolidae) on Gills of Bighead Carp *Hypophthalmichthys nobilis* in China

**DOI:** 10.3390/pathogens12040562

**Published:** 2023-04-06

**Authors:** Lin Lin, Houda Cheng, Wenxiang Li, Ming Li, Hong Zou, Guitang Wang

**Affiliations:** 1 College of Fisheries, Huazhong Agricultural University, Wuhan 430070, China; 2 State Key Laboratory of Freshwater Ecology and Biotechnology and Key Laboratory of Aquaculture Disease Control, Ministry of Agriculture, Institute of Hydrobiology, Chinese Academy of Sciences, Wuhan 430072, China; 3 College of Advanced Agricultural Science, University of Chinese Academy of Sciences, Beijing 100049, China

**Keywords:** fish leech, species identification, *Hypophthalmichthys nobilis*, *Limnotrachelobdella hypophthalmichthysa*

## Abstract

We describe the characterization of a novel fish leech species found on the gills of bighead carp (*Hypophthalmichthys nobilis*) from lakes and reservoirs in China. This leech is morphologically similar to *Limnotrachelobdella sinensis* recorded on goldfish and common carp. However, there are 0–2 pairs of symmetrical or asymmetrical eyes and 10 pairs of pulsatile vesicles in the newly discovered leech, in remarkable contrast to *L. sinensis*. Except for bighead carp, where it demonstrated a higher than 90% prevalence, and silver carp (*H. molitrix*), where there was low infection, this leech was not detected on any other fish from the Qiandao reservoir in China that were examined during this investigation. Molecular analyses indicated 87.8% ITS sequence identity with *L. sinensis* and 85.0 and 86.1% COX1 sequence identity with *L. sinensis* and *L. okae*, respectively. The uncorrected p-distance based on the COX1 sequence was found to be 15.1 and 14.0% for *L. sinensis* and *L. okae*, respectively, suggesting interspecific variation. Phylogenetic analyses based on the combination of 18S and COX1 sequences showed that the newly discovered leech groups with *Limnotrachelobdella* species. Histopathological observation indicated that attachment of the leech on the gill rakers and gill arches causes a loss of connective tissue, hemorrhage, and ulceration. Based on the morphology, molecular analyses, and host specificity, we conclude that this leech is a new species of *Limnotrachelobdella* and named it *Limnotrachelobdella hypophthalmichthysa* n. sp.

## 1. Introduction

Leeches (Annelida: Hirudinida) are usually found in freshwater, estuarine, terrestrial, and marine ecosystems [1]. Species of leeches of the genera *Piscicola* and *Limnotrachelobdella* (Rhynchobdellida: Piscicolidae) tend to parasitize marine and freshwater fish [2,3,4,5]. Fish leeches have been suggested as pathogens to hosts [6], causing severe epidermal erosion on the attachment and secondary infection of viruses or bacteria [5,7]. The heavy infestation of leeches can even cause the mortality of hosts [8,9].

It has traditionally been accepted that there are three subfamily members in Piscicolidae: Piscicolinae Johnston, 1865; Platybdellinae Epshtein, 1970; and Pontobdellinae Llewellyn, 1966. However, according to the latest phylogenetic analyses, Piscicolinae and Platybdellinae are both now considered to be polyphyletic groups based on their nuclear and mitochondrial gene sequences and morphology [10,11]. *Limnotrachelobdella*, belonging to Piscicolidae, comprises five nominal species: *L. sinensis* (Blanchard, 1896) [12], *L. okae* (Moore, 1924) [13], *L. taimeni* (Epstein 1957) [14], *L. fujianensis* (Yang, 1987) [15], and *L. turkestanica* (Stschegolew, 1912) [16]. The first four species have been previously found in China [17].

In recent years, a kind of leech was only found on the bighead carp, *Hypophthalmichthys nobilis* (Richardson, 1845), in reservoirs and lakes in China. A preliminary investigation showed that this leech is morphologically similar to *L. sinensis*, which has been recorded on goldfish (*Carassius auratus*) and common carp *(Cyprinus carpio)* in China [15]. However, the leech found on bighead carp is distinct from *L. sinensis* and has not been found on goldfish or common carp during investigations. Therefore, the leech on bighead carp was identified by its morphological characteristics and further characterized based on molecular sequence analyses as well histopathological observation.

## 2. Materials and Methods

### 2.1. Sample Collection and Preservation

Fish were collected for the examination of leeches in the Qiandao reservoir (29°33′44.32″ N, 119°01′51.98″ E), Zhejiang province, China, during 2021–2022. Leeches were detached from fish gills using forceps. Living leeches were stocked in fresh water for morphological analyses. Some leeches were preserved in 100% ethanol and stored at −20 °C for DNA extraction, including one specimen each from Hubei and Jiangxi provinces. In addition, gills infected with leeches were preserved in 4% paraformaldehyde for histopathological observation.

### 2.2. Morphological Identification

First, living leeches were examined for the number of eyes within 48 h after detachment from gills. Then, specimens were anesthetized by adding ethanol in a dropwise manner for a subsequent observation of the digestive and reproductive system under a stereomicroscope. The epidermis of the slack leech was separated using a scalpel applied along the longitudinal centerline dorsally or ventrally. The ventral nerve cord was then separated from the epidermis using ophthalmic forceps to determine its positional relationship to the reproductive and digestive systems. The digestive system was directly observed in small leeches collected from December to February when it has thinner and more transparent muscle layers. In addition, specimens fixed in 4% paraformaldehyde were photographed using a Panasonic DMC-GX85CGK digital camera. The materials were deposited in the collection of the Institute of Hydrobiology, Chinese Academy of Science, Hubei, China [Holotype (accession no. LH-Z3) and four paratypes (accession nos. LH-Z2, Z4, H1, and J1)].

### 2.3. Histopathological Observation

Gills fixed in 4% paraformaldehyde were trimmed into 1 cm × 1 cm tissue blocks and dehydrated using a series of graded ethanol solutions and cleared in xylene. After the paraffin wax solidified, 4 μm paraffin sections were taken and mounted on slides for hematoxylin and eosin (H and E) staining. The slides were subsequently examined under a stereomicroscope.

### 2.4. DNA Extraction, Amplification, and Sequencing

Muscles of the lateral body and caudal sucker were removed for DNA extraction to avoid the presence of host blood in the leech gut. Genomic DNA was isolated using an Aidlab Genomic DNA extraction kit (Aidlab Co., Beijing, China) according to the manufacturer’s instructions. Ribosomal encoded genes, including the 18S DNA gene, internal transcribed spacer 1–5.8S rDNA—internal transcribed spacer 2 (ITS) and fragments of the mitochondrial gene, cytochrome c oxidase subunit I (COX1), were amplified from the genomic DNA. All the genes used, following polymerase chain reaction (PCR) condition, were according to the manufacturer’s instructions (Takara Bio, China): 98 °C for 2 min, 40 cycles of 98 °C for 10 s, 50 °C for 15 s, and 68 °C for 1 min, and final extension at 68 °C for 10 min. Primer pairs were designed according to the sequence in similar species (Table 1). PCR products were sequenced using an ABI 3730 automatic sequencer (Sanger Sequencing). The sequences were assembled manually using DNAstar software v7.1 (Madison, Wisconsin, USA).

### 2.5. Molecular Identification and Phylogenetic Analyses

Fish were collected for leech examination in the Qiandao reservoir (29°33′44.32″ N, 119°01′51.98″ E), Zhejiang province, China, during 2021–2022. For molecular analyses, ITS and COX1 were chosen to compare specimens collected from three places: Zhejiang, Hubei, and Jiangxi provinces. In addition, the new species’ similarity to *L. sinensis* and *L. okae* was verified in the NCBI GenBank database. The uncorrected p-distance was used to examine COX1 genetic variation among *Limnotrachelobdella* species with 1000 replicates in MEGA11 [18].

Phylogenetic analyses were performed, combining 18S and COX1 gene datasets using 29 sequences of Piscicolidae leech species from GenBank (Table 2). All of the sequences were imported into PhyloSuite [19] and aligned using MAFFT 7.149 [20] with auto strategy and normal alignment mode; further adjustments to sequence alignments were manually applied. Then, the 18S and COX1 sequences were concatenated using the “concatenate sequence” function in PhyloSuite. The best partitioning scheme and evolutionary models for 2 predefined partitions were selected using PartitionFinder2 [21] with a greedy algorithm. Bayesian inference (BI) phylogenies were inferred using MrBayes 3.2.6 [22] under the partition model (2 parallel runs, 100,000 generations), with the initial 25% of sampled data discarded as burn-in and sampling frequency of 3000.

## 3. Results

### 3.1. Infection by Fish Leeches

In total, 16 species of fish from the Qiandao reservoir were examined, including *Carassius auratus*, *Megalobrama amblycephala*, and *Opsariichthys bidens*. Fish leeches were found on bighead carp at higher than 90% prevalence and silver carp (*Hypophthalmichthys molitrix*) at extremely low prevalence (only one silver carp infected with one leech).

### 3.2. Description

**Figure 1:**
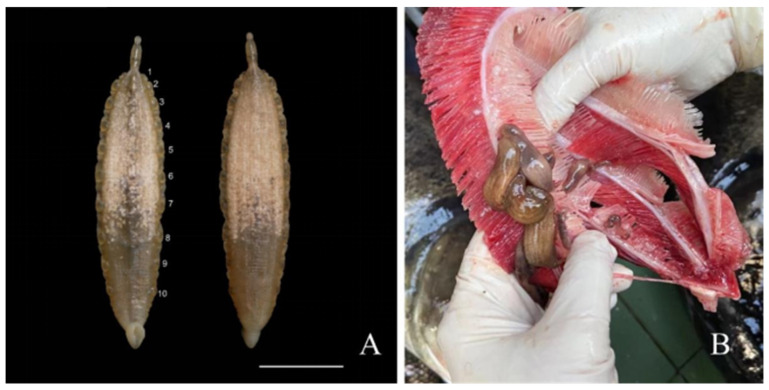
Whole view of preserved and fresh *Limnotrachelobdella hypophthalmichthysa* n. sp. (**A**) Specimen preserved in 4% paraformaldehyde, ventral (left) and dorsal (right) view. Numbers indicate 10 pairs of pulsatile vesicles. Scale bar, 1 cm. (**B**) Fresh *L. hypophthalmichthysa* n. sp. attached to gill rakers and arches of bighead carp *Hypophthalmichthys nobilis*.

**Figure 2:**
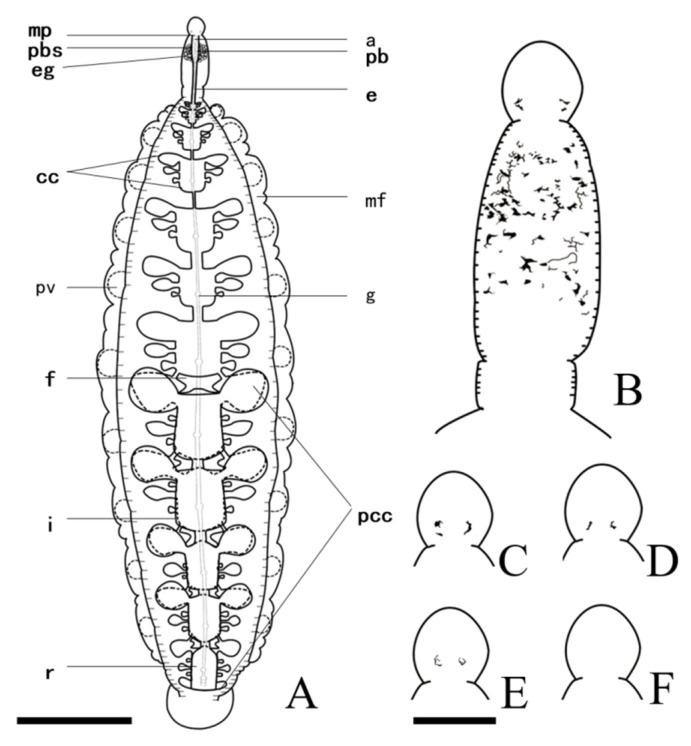
Digestive system, eyes, and pigment of *Limnotrachelobdella hypophthalmichthysa* n. sp. (**A**) Digestive system and overall structure. Patterns of eyes and pigment: (**B**) 2 pairs of eyes, (**C**) right eyes of 2 pairs slightly fused, (**D**) 1 pair of eyes, (**E**) degenerated eyes, and (**F**) no eyes (**F**). Abbreviations: cc, crop chamber; e, esophagus; eg, esophageal gland; f, fenestrae; g, ganglion of ventral nerve cord; a, anterior nerve mass; i, intestine; mf, margin fold; mp, mouthpore; pb, proboscis; pcc, posterior crop caeca; r, rectum; pbs, proboscis sheath; pv, pulsatile vesicle. Parts of digestive system shown in bold type. Scale bar: A, 1 cm; B–F; 2 mm.

External morphology: The body distinctly divides into trachelosome and urosome, with a total length of 4.6–70.3 mm and width of 0.9–16.2 mm (Figure 1A). Body skin is smooth, but with numerous brown pigments on the surface of the trachelosome region (Figure 2B). Body color of the living leech is reddish or reddish brown (Figure 1B). Eyes, 0–2 pairs, symmetrical or asymmetrical or degenerated eyes on oral sucker (Figure 2B–F). Clitellum is ring-like, sharply distinct from pre-clitellum and urosome. In the urosome region, the body musculature is well developed. Complete somite is composed of six annuli, but the external appearance suggests it is divided into fourteen annuli, which is consistent with *L. sinensis*. Grape-like tissue is present in the area before somite XXII in the mature individuals. Pulsatile vesicles are of 10 pairs, present separately on each somite of somites XIV–XXIII and matched against sequential numbers of the ventral nerve cord ganglion, respectively. Caudal sucker is deep cup-like, thick, and ventrally directed, and its diameter is smaller than the maximum body width. Anal is separated by the last two annuli of somite XXVII and is difficult to be observed. 

**Figure 3:**
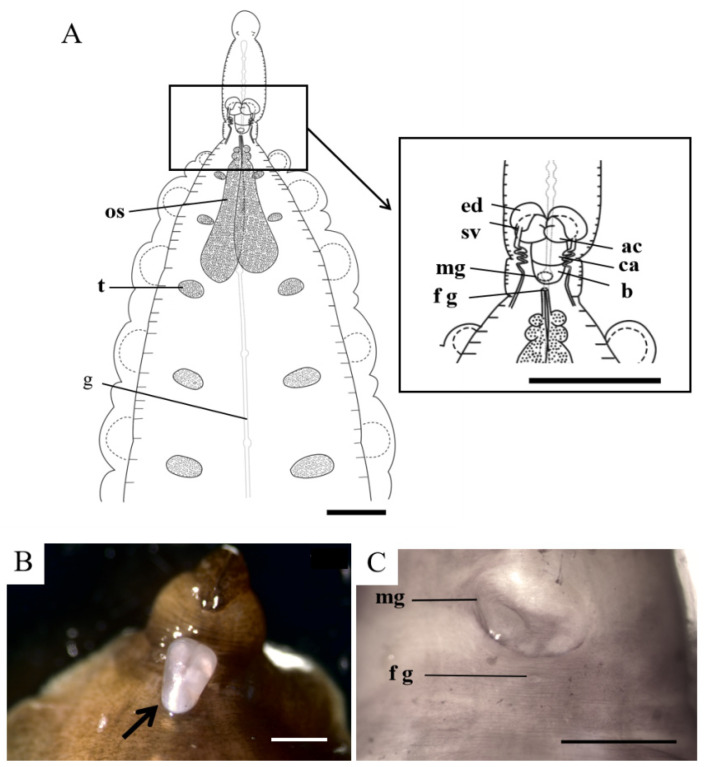
Reproductive system of *Limnotrachelobdella hypophthalmichthysa* n. sp. (**A**) Overall dorsal view of reproductive system (left) and reproductive organs in trachelosome region (right). (**B**) Ejaculatory duct, atrial cornua, and common atrium, wrapped by bursa, evert out of male gonopore (arrow). (**C**) Male and female gonopores. Abbreviations: ac, atrial cornua; b, bursa; ca, common atrium; ed, ejaculatory duct; fg, female gonopore; g, ganglion of ventral nerve cord; os, ovisac; mp, male gonopore; t, testisac; sv, seminal vesicle. Part of reproductive system shown in bold type. Scale bars: A, 3 mm; B, 2 mm; C, 1 mm.

Internal morphology: Esophagus passes posteriorly from the proboscis to somite XI. Crop extends from somite XIII, and with seven chambers, each with three pairs of pouches (the first two bigger than the last). Posterior crop caeca fused with five fenestraes, forming five well-developed chambers similar to crop chambers. Intestine consists of four chambers without lateral pouches (Figure 2A). Male gonopore is large, round and crinkled. Female gonopore is below the male gonopore, particularly smaller, and elliptical (Figure 3C). Because of the smooth skin of the clitellum, it was difficult to determine the number of annuli between the two gonopores. Testisacs are five pairs, elliptical in shape, located on somites XIV–XVIII and alternating with crop caeca, slightly posterior to the ganglion of the ventral nerve cord (Figure 3A). Ejaculatory duct and atrial cornua are both in kidney-like shape. Accessory gland cells are absent. Common atrium is near-spherical, connecting a thick and large bursa that leads to the male gonopore. The ejaculatory duct, atrial cornua, and common atrium are all semitranslucent and threaded before fixation and are often wrapped by the brusa, then evert out of the male gonopore (Figure 3B). Conducting tissue and vector tissue are absent. Paired ovisacs are sac-like, extending to somite XV. Each ovisac consists of three lobes with the third lobe the largest.

### 3.3. Molecular Identification and Phylogenetic Analyses

From the three specimens (Z1, H1 and J1), the 18S rDNA (1712–1713 bp), ITS (1489–1495 bp), and COX1 (1537 bp) genes sequence were obtained. Molecular analyses indicate that the ITS and COX1 sequence identity of the three specimens (collected from Zhejiang, Hubei, and Jiangxi) ranges from 98.6 to 99.7%. The new leech shares 87.8% of ITS sequence identity with *L. sinensis* and 85.0 and 86.1% of COX1 sequence identity with *L. sinensis* and *L. okae*, respectively. The uncorrected p-distance of COX1 sequence was found to be 0.3% for the three specimens in pairwise comparison, and 15.1 and 14.1% with *L. sinensis* and *L. okae*, respectively. 

**Figure 4:**
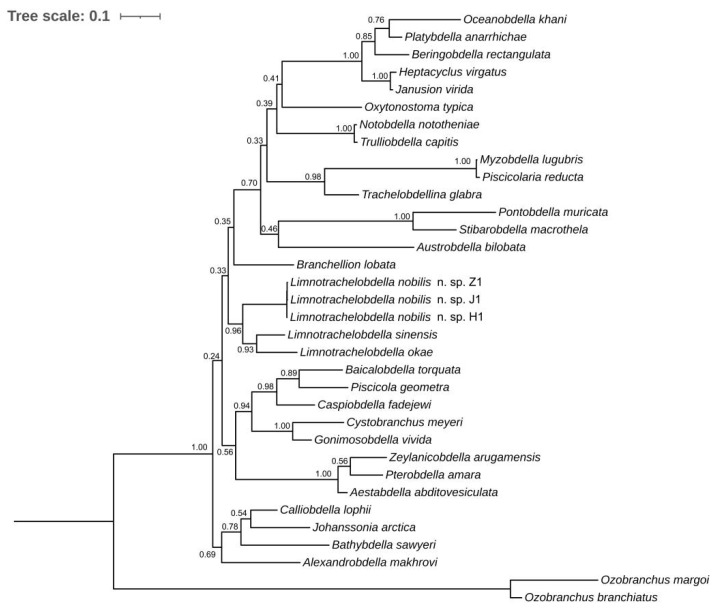
BI tree resulting from the analyses of combined 18S rDNA and COX1 data containing *Limnotrachelobdella hypophthalmichthysa* n. sp. and related species of the Piscicolidae family. *Ozobranchus margoi* and *O. branchiatus*, of the same suborder, and Oceanobdelliformes are chosen as outgroups. Node numbers represent Bayesian posterior probabilities. *L. hypophthalmichthysa* n. sp. specimens were collected from three places (Z1 from Zhejiang, H1 from Hubei, J1 from Jiangxi).

The phylogenetic tree produced by BI analyses of a combination of 18S and COX1 sequences indicates that *L. hypophthalmichthysa* n. sp. groups with *L. sinensis* and *L. okae* (Figure 4).

### 3.4. Taxonomic Summary

Family: Piscicolidae Johnston, 1865.

Genus: *Limnotrachelobdella* Epshtein, 1968.

Species: *Limnotrachelobdella hypophthalmichthysa* n. sp.

Type host: *Hypophthalmichthys nobilis* (Richardson, 1845).

Type locality: Qiandao reservoir (29°33′44.32″ N, 119°01′51.98″ E), Zhejiang province, China.

Type material: Holotype (accession no. LH-Z3) and four paratypes (accession nos. LH-Z2, Z4, H1, and J1) were deposited in the Museum of the Institute of Hydrobiology, Chinese Academy of Sciences, China.

Infection site on host: Gill arch and gill raker.

ZooBank registration: The Life Science Identifier (LSID) for *Limnotrachelobdella hypophthalmichthysa* n. sp. is urn:lsid:zoobank.org:act:5996B408-EB5E-4470-974C-30962DF5D041.

Etymology: The species name is derived from the genus name of fish hosts *Hypophthalmichthys nobilis* (Richardson, 1845) and *Hypophthalmichthys molitrix* (Valenciennes, 1844).

### 3.5. Histopathological Analyses

**Figure 5:**
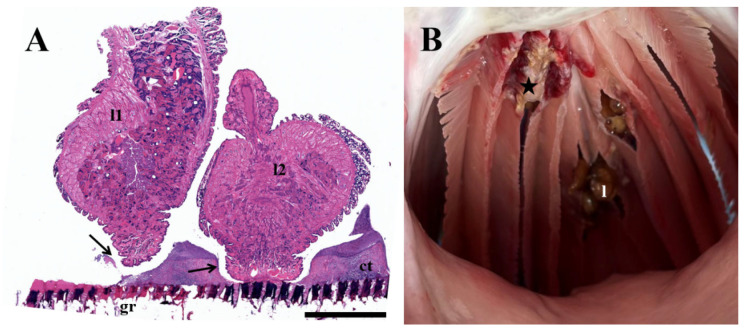
Histopathology of bighead carp *Hypophthalmichthys nobilis* parasitized by *Limnotrachelobdella hypophthalmichthysa* n. sp. (**A**) Gill raker section (H and E stained) infected by two *L. hypophthalmichthysa* n. sp. (l1, l2), with eroded connective tissue (arrows). Scale bar: 200 mm. (**B**) Ulceration and hemorrhage at attachment site of leeches (star). Abbreviations: ct, connective tissue; gr, gill raker; l, leech.

Attachment of the leeches on gill rakers and gill arches with the caudal sucker causes loss of connective tissue (Figure 5A). Hemorrhage and ulceration were also observed on gills infected by leeches (Figure 5B).

## 4. Discussion

Yang [17] presented identification keys for the genus *Limnotrachelobdella*: the distinction between trachelosome and urosome, deeply cup-shaped caudal sucker with a smaller diameter than the maximum width of the body, six annuli in each complete somite, ten to thirteen pairs of pulsatile vesicles on the urosome, a fused posterior crop caeca, and five to six pairs of testisacs. The morphological identification of the fish leeches we collected revealed conformance to these features. So far, five nominal species of *Limnotrachelobdella* have been recorded: *L. sinensis* (Blanchard, 1896), *L. okae* (Moore, 1924), *L. taimeni* (Epshtein, 1957), *L. fujianensis* (Yang, 1987), and *L. turkestanica* (Stschegolew, 1912). Owing to its similar appearance, fish leech *L. hypophthalmichthysa* n. sp. collected from bighead carp can be easily mistaken for *L. sinensis*. However, it has 10 pairs of pulsatile vesicles, while the latter has 11 pairs. Meanwhile, the following characteristics are helpful in distinguishing it from five known species: no eyes or one to two pairs of symmetrical, asymmetrical, or degenerated eyes, seven pairs of crop caeca, and five pairs of testisacs (Table 3). In addition, there are numerous brown pigments on the surface of the trachelosome region, and the appearance of the atrial cornua, which extends dorsally, is typical.

*L. hypophthalmichthysa* n. sp. is also different from other *Limnotrachelobdella* species in terms of its host range. *L. sinensis* has been recorded on goldfish/silver crucian carp such as *Carassius auratus* and *C. cuvieri* in Korea [23,24,25], *C. cuvieri* and *C. auratus langsdorfii* in Japan [7,26], and *C. gibelio* in Russia [17,27]. *L. sinensis* has also been found on common carp such as *Cyprinus carpio* in China [15] and Japan [28] and *C. carpio haematopterus* in Russia [20,27]. Meanwhile, *L. taimeni* was found on *Hucho taimen* (Pallas, 1773) [14] and *L. fujianensis* was found on *Epinephelus akaara* (Temminck and Schlegel, 1842) and *Planiliza affinis* (Gunther, 1861) [15,17], respectively. However, *L. okae* was recorded on fish in the Acipenseridae, Cyprinidae, Salmonidae, Lateolabracidae, Carangidae, Paralichthyidae, and Tetraodontidae families [29,30], exhibiting a wide host range. *L. turkestanica* was also considered to have a wide range of fish hosts [31,32]. Therefore, unlike *L. okae* and *L. turkestanica*, *L. sinensis*, *L. taimeni*, and *L. fujianensis* are fish leeches with high host specificity. *L. hypophthalmichthysa* n. sp. has only been found on *Hypophthalmichthys nobilis* and *H. molitrix*, which suggests that it is a highly host-specific species. In addition, *L. sinensis* was found on the operculums of goldfish and common carp [7,26,28], whereas the leech representing the focus of this study has been found on gill arches and gill rakers of bighead carp. Therefore, its different host species and infection sites compared with *L. sinensis* suggests that this leech is a new species.

The ITS sequences of the species in genus *Whitmania* (Hirudinida: Haemopidae) (*W. acranulata*, JX885692; *W. laevis*, JX885693; *W. plgra*, EU652726) was obtained on Genbank database and the 92.7 to 98.4% of sequence identity could be calculated easily. In this study, the high ITS sequence identity of 98.6 to 99.5% of the specimens from Zhejiang, Hubei, and Jiangxi indicates that they are of the same species. However, only 87.8% ITS sequence identity was found between the new leech and *L. sinensis*, which was smaller than the range of interspecific divergence in genus *Whitmania*. Based on the COX1 sequence, the uncorrected p-distance is 15.1% with *L. sinensis* and 14.0% with *L. okae*, which are higher than the intraspecific divergence of 0.3% for specimens of the new leech from Zhejiang, Hubei, and Jiangxi. For COX1, a 2.0% genetic distance was found to be indicative of intraspecific divergence [33,34,35], with interspecific divergence of 13.3 to 23.6% between *Helobdella blinni* (Hirudinidea: Glossiphoniidae) and other species in the genus *Helobdella* [36]. In the present study, the p-distance of 14.0 to 15.1% is within this range of interspecific divergence. Therefore, the low ITS sequence identity and high genetic distance between the new leech and other species in *Limnotrachelobdella* provides molecular evidence that the leech found on bighead carp is a novel species of the genus *Limnotrachelobdella*. 

Williams and Burreson [11] attempted to sequence 18S rDNA, COX1, and ND1 gene data of a large number of species in Piscicolidae and illuminated the phylogeny of the family by considering the sequences above in combination with morphological data. In this study, the BI tree is consistent with our expectations, implying that *L. hypophthalmichthysa* n. sp. groups together with *L. sinensis* and *L. okae*. 

In summary, based on the morphology, molecular analyses, host specificity, and infection site, the leech infecting bighead carp is a novel species of *Limnotrachelobdella*, which we named *Limnotrachelobdella hypophthalmichthysa* n. sp.

## Figures and Tables

**Table 1:** List of the primers and accession numbers of genes used in this study.

Gene or Region	Primer Name	Sequence (5′-3′)	Accession Number (Gene Length)		
			*Limnotrachelobdella hypophthalmichthysa* n. sp.		
			Z1	H1	J1
18S	18SF	5′-GATTAAGCCATGCATGTCTA-3′	OP595730 (1712 bp)	OP709950 (1712 bp)	OP709959 (1713 bp)
	18SR	5′-ACTTCCTCTAGATGATCAAG-3′			
ITS	ITSF	5′-TCGCGTTGATTACGTCCCTG-3′	OP723914 (1495 bp)	OP723913 (1490 bp)	OP723912 (1489 bp)
	ITSR	5′-GCATTCTCAAACAACCCGAC-3′			
COX1	COX1F	5′-GCTTCTAACTTTTAGTRGRTAG-3′	OP711958 (1537 bp)	OP712182 (1537 bp)	OP712183 (1537 bp)
	COX1R	5′-CTTCTARTTAACAGTTAGRTGCA-3′			

**Table 2:** Species used in phylogenetic analyses with details of their environment and GenBank accession number. *L. hypophthalmichthysa* n. sp. Z1, H1, and J1 were collected from Zhejiang, Hubei, and Jiangxi, respectively.

Species	Environment	GenBank Accession Number	
		18S	COX1
Ingroup			
Piscicolidae			
*Stibarobdella macrothela*	Brackish to marine	DQ414295	DQ414340
*Pontobdella muricata*	Marine	AF099945	AY336029
*Aestabdella abditovesiculata*	Marine	DQ414254	DQ414300
*Austrobdella bilobata*	Marine	DQ414255	DQ414301
*Bathybdella sawyeri*	Marine	DQ414265	DQ414311
*Beringobdella rectangulata*	Marine	DQ414264	DQ414310
*Heptacyclus virgatus*	Brackish to marine	DQ414273	DQ414319
*Janusion virida*	Marine	DQ414281	N/A
*Myzobdella lugubris*	Freshwater to marine	DQ414278	DQ414324
*Notobdella nototheniae*	Marine	DQ414283	DQ414328
*Oceanobdella khani*	Marine	DQ414286	DQ414331
*Oxytonostoma typica*	Marine	DQ414288	DQ414333
*Piscicolaria reducta*	Freshwater	DQ414294	DQ414339
*Platybdella anarrhichae*	Marine	DQ414290	DQ414335
*Pterobdella amara*	Brackish	DQ414289	DQ414334
*Trulliobdella capitis*	Marine	N/A	AY336030
*Zeylanicobdella arugamensis*	Brackish to marine	DQ414299	DQ414344
*Alexandrobdella makhrovi*	Freshwater	MN312187	MN295413
*Baicalobdella torquata*	Freshwater	N/A	AY336018
*Branchellion lobata*	Marine	DQ414261	DQ414307
*Calliobdella lophii*	Marine	DQ414268	DQ414314
*Caspiobdella fadejewi*	Freshwater	N/A	AY336019
*Cystobranchus meyeri*	Freshwater	DQ414269	DQ414315
*Gonimosobdella vivida*	Freshwater	AF115992	AF003260
*Johanssonia arctica*	Marine	DQ414274	DQ414320
*Limnotrachelobdella hypophthalmichthysa* n. sp. Z1	Freshwater	OP595730	OP711958
*Limnotrachelobdella hypophthalmichthysa* n. sp. H1		OP709950	OP712182
*Limnotrachelobdella hypophthalmichthysa* n. sp. J1		OP709959	OP712183
*Limnotrachelobdella okae*	Freshwater to marine	N/A	AY336022
*Limnotrachelobdella sinensis*	Freshwater to brackish	LC275139	LC275140
*Piscicola geometra*	Freshwater to marine	AF115995	AF003280
*Trachelobdellina glabra*	Marine	N/A	EF405597
Outgroup			
Ozobranchidae			
*Ozobranchus branchiatus*	Marine	KF728214	KF728213
*Ozobranchus margoi*	Marine	AF115991	AF003268

**Table 3:** Comparison of morphological differences among six species of *Limnotrachelobdella*.

	*L. hypophthalmichthysa* n. sp.	*L. sinensis*	*L. okae*	*L. taimeni*	*L. fujianensis*	*L. turkestanica*
Eyes	0–2	2	2	0 *	2	N/A
Pulsatile vesicles	10	11	11–13	10	12	11
Testisacs	5	6	6	N/A	6	5

* Uncertain. N/A, not available.

## Data Availability

Data are available from the authors upon reasonable request.
